# Implementation of the Patient Counselling Service at the Cancer Hospital in Radom, Poland

**DOI:** 10.3390/ijerph192013642

**Published:** 2022-10-21

**Authors:** Katarzyna Grzyb, Martyna Meresińska, Urszula Religioni, Grzegorz Juszczyk, Jakub Płaczek, Agnieszka Neumann-Podczaska, Filip M. Szymański, Beata Chełstowska, Katarzyna Wieczorowska-Tobis, Szczepan Cofta, Sławomir Tobis, Rafał Staszewski, Regis Vaillancourt, Rafał Majewski, Justyna Hernik, Katarina Fehir Sola, Eliza Blicharska, Justyna Kaźmierczak, Ewa Rutkowska, Elżbieta Prygiel, Monika Skierska, Monika Nawara, Izabela Korbiewska, Jerzy Krysiński, Piotr Merks

**Affiliations:** 1 Radom Oncology Center im, Bohaterów Radomskiego Czerwca 76′, 26-600 Radom, Poland; 2 Department of Pharmaceutical Technology, Faculty of Pharmacy, Collegium Medicum in Bydgoszcz, 85-089 Bydgoszcz, Poland; 3 School of Public Health, Centre of Postgraduate Medical Education of Warsaw, Kleczewska 61/63, 01-826 Warsaw, Poland; 4 Department of Public Health, Medical University of Warsaw, 02-097 Warsaw, Poland; 5 Department of Palliative Medicine, Poznan University of Medical Sciences, 61-245 Poznan, Poland; 6 Department of Civilization Diseases, Faculty of Medicine, Collegium Medicum, Cardinal Stefan Wyszynski University in Warsaw, 01-938 Warsaw, Poland; 7 Department of Biochemistry and Laboratory Diagnostics, Faculty of Medicine, Collegium Medicum, Cardinal Stefan Wyszynski University in Warsaw, 01-938 Warsaw, Poland; 8 Heliodor Swiecicki Clinical Hospital in Poznan, 60-355 Poznan, Poland; 9 Department of Pulmonology, Allergology and Respiratory Oncology, Poznan University of Medical Sciences, 60-569 Poznan, Poland; 10 Department of Occupational Therapy, Poznan University of Medical Sciences, 60-781 Poznan, Poland; 11 Department of Hypertension, Angiology and Internal Medicine, Poznan University of Medical Sciences, 61-848 Poznań, Poland; 12 Department of Pharmacology and Clinical Pharmacology, Faculty of Medicine, Collegium Medicum, Cardinal Stefan Wyszynski University in Warsaw, 01-938 Warszawa, Poland; 13 Pharmacy of Bjelovar, Petra Preradovića, 43000 Bjelovar, Croatia; 14 Department of Analytical Chemistry, Medical University of Lublin, Chodźki 4a, 20-093 Lublin, Poland; 15 Zdrowit sp. z o.o., Pharmacy Chain, 41-940 Piekary Śląskie, Poland; 16 Rehabilitation Faculty of Medical Sciences, Medical University of Warsaw, Żwirki i Wigury 61, 02-091 Warsaw, Poland

**Keywords:** chronic disease, cancer, oncology, hospital pharmacy, pharmacist, adherence

## Abstract

**Background:** Non-adherence occurs in various groups of patients, including those with chronic diseases. One strategy to increase adherence among oncological patients is to individualise treatment and expand pharmaceutical care. Pharmaceutical labels that remind patients how they should take their medications are of great importance in this respect. **Objective:** The main objective of this study was to evaluate medication adherence in oncological patients, and to gather their opinions on the individual medication labelling system as an element of effective treatment. **Methods:** The study was conducted in 2021 among 82 patients of the oncological department of the Centre of Oncology in Radom. The research tool was a questionnaire consisting of personal data and two parts relating to the patient’s disease and the medication labelling system. **Results:** Nearly half of the respondents reported that they forget to take medications and how they should take them. These problems increased with the age of the patient and the number of administered medications. Of the respondents, 89% stated that the labels with dosing information are helpful. Over 67% agreed that these labels should be affixed to all medications. Nearly 90% of the respondents believed the labels should be available in all pharmacies. **Conclusions:** Non-adherence is a common phenomenon among oncological patients. Pharmacists providing a labelling service for medicinal products can play a significant role in reducing this phenomenon.

## 1. Introduction

In recent years, non-adherence among chronic patients has become increasingly worrisome [1,2]. There are many reasons for non-adherence, such as factors connected with the patients and their socioeconomic situation, as well as with the organisation of healthcare systems and patient relationships with representatives of therapeutic teams [3,4].

According to the World Health Organisation (WHO), improving treatment adherence is a contemporary challenge for many healthcare systems. One solution is the implementation of interventions adjusted to the individual needs of patients, which means that healthcare systems and their service providers need to develop methods to evaluate adherence and the factors influencing adherence. Therapeutic processes are long and dynamic; therefore, assessment of the level of adherence should undergo regular evaluation [5].

Oncological diseases pose a serious challenge for healthcare systems, being the second largest cause of deaths in Poland and many other developed countries [6]. Due to the specificity of malignant tumours, their invasive character and non-controlled growth, their treatment is demanding and often based on taking radical measures with side effects. Both surgical and systemic treatment impact the body; therefore, oncological therapies bear a high risk of adverse effects [7,8].

In patient treatment, such as in oncological diseases, increasing attention is being paid to the role of pharmacists as members of an interdisciplinary therapeutic team. Thanks to their knowledge and broad competencies, in particular with regard to the knowledge of the use of medicines, pharmacists can actively take part in patient treatment, contributing to increased adherence and better treatment outcomes [9,10].

One strategy to increase adherence among oncological patients is to individualise treatment and expand pharmaceutical care [11]. This care should include pharmaceutical consultations and adjusting treatment to patients’ needs and expectations.

The role of pharmacists is particularly demanding in the context of pharmaceutical care of oncological patients [10]. Chemotherapy is characterised by high toxicity and multiple side effects. These medications often interact with each other, which can increase the frequency and severity of adverse effects. Pharmaceutical consultations permit adjusting optimal treatments, eliminating possible interactions, thus increasing treatment tolerance [12,13]. Individual pharmaceutical care could also encompass the implementation of special pharmaceutical labels that would help patients take their medications properly. Studies indicate that pharmaceutical labels make information about medications more accessible and improve understanding and remembering information about medication use [14,15].

Thus, the objective of this study was to evaluate medication adherence by oncological patients, and to gather opinions on the individual medication labelling system as an element of effective treatment. The study also aimed to determine factors that can have a significant influence on adherence. The respondents’ demographic characteristics and previous experiences with treatment were taken into consideration.

## 2. Methods

The study was conducted in 2021 at the oncology department of the Cancer Hospital in Radom.

The research tool was a questionnaire consisting of personal data and two further parts. The first part contained questions regarding chronic diseases, the number of administered medications and adherence in home conditions. The second part evaluated the Individual Medication Labelling System. All the questions were single-choice closed questions.

The respondents continued their treatment with their prescribed medicinal products in home conditions, following discharge from the hospital.

During medical consultations, the patients were given prescriptions for cancer medications dispensed from the hospital pharmacy. They were then invited to a consultation with a pharmacist who talked to them and provided them with information about the prescribed medications, their potential adverse effects and ways of coping with these. After the consultation, the patients received their medications and signed the receipt protocol. The patients had the possibility to ask questions regarding the prescribed medicinal products. At the end of the meeting, the patients filled in the questionnaire in paper form regarding their satisfaction with the pharmaceutical consultation, which was tantamount to giving their consent to participate in the study.

The study was approved by the Bioethics Committee of the Nicolaus Copernicus University in Toruń at the Ludwik Rydygier Medical College in Bydgoszcz, Poland (KB 524/2021).

The data obtained through the questionnaire were entered into a spreadsheet for quantitative and statistical analysis to verify the research hypotheses. The statistical analysis was performed using PSPP software. Due to the nominal character of the variables, Pearson chi-square tests were performed to verify the hypotheses. The level of statistical significance was taken at *p* = 0.05* (Supplementary materials).

## 3. Results

### 3.1. Characteristics of the Study Group

A total of 82 patients participated in the study, of which 58.5% were women and 41.5% were men. The study group was characterised by a wide age variation. The largest groups (24.4%) were aged 50–59 years and 60+ years. A smaller group aged 40–49 years constituted 23.2%. Nearly every fifth respondent (19.5%) was 30–39 years old. The smallest group consisted of young patients aged 20–29 and 16–19 years, 3.7% and 4.9%, respectively.

Of the respondents, 13.4% had an elementary education, and 14.6% had a basic vocational education. The largest group (37.8%) had secondary education and the remaining 34.1% had higher education.

The respondents most often declared doing intellectual work (32.9%). Every fourth respondent was a physical worker (25.6%), and every fifth respondent was eligible for a retirement pension or disability pension (19.5%). Healthcare professionals constituted 4.9%, and entrepreneurs 12.2%. A small proportion was pupils and students (4.9%).

In addition to oncological diseases, the vast majority of the respondents (90.2%) suffered from chronic comorbidities (for example, cardiovascular disease, diabetes), and along with the medications dispensed at the hospital pharmacy also took medications for those chronic conditions. One in ten respondents had never taken any medications for long-term conditions.

The patients most frequently took 3–5 long-term medicinal products (29.3%): 14.6% of the respondents were administered six or more long-term medications, one in ten respondents (9.8%) received two medications, and 7.3% received one medication.

### 3.2. Patient Medication Adherence

First, the respondents were asked about receiving written instructions from their physician; 75.6% of the respondents gave positive answers. Then, they were asked if they sometimes lost these written instructions, which was confirmed by half of the respondents (52.4%).

Their medication-related issues were also sought. They more often forgot to take their medications (15.9%) than how to take them (9.8%). More than one-third of the respondents (35.4%) declared that they sometimes forgot to take their medications, and 40.2% of them sometimes forgot how to take them. Of the respondents, 39% reported that they seldom forgot to take medications and how to take them. One in ten respondents (9.8%) never forgot to take their medications, and 11% always remembered how to take them.

Demographic characteristics such as sex, age, education, the kind of work and the number of administered medications had an influence on forgetting to take medications by the respondents and are shown in Table 1.

The conducted analysis indicates that forgetting to take medications is connected with age (*p* = 0.000), the kind of professional activity (*p* = 0.000) and the number of administered medications (*p* = 0.003). The analysis by age shows that young patients (16–19 and 20–29 years old) seldom forgot to take their medications. The percentage of respondents who frequently forgot to take their medications was highest among those aged 60 years and older (nearly 50%). Almost half (40%) of the oldest respondents declared that they sometimes forgot to take their medications, but none of them reported problems with taking their medications.

When analysing the relationship between the level of education and forgetting to take medications, it was noticed that healthcare professionals, pupils and students had the least problems with forgetting. Intellectual (office) workers did not report forgetting to take their medications either. Most frequently, retirees and pensioners forgot to take their medications.

As far as the number of administered medications is concerned, the respondents who took more than five medicinal products had the biggest problems with remembering how they should take their medications. This problem was least severe among the respondents who took one long-term medication.

In order to verify if the respondents had sufficient knowledge on how to take their medications, they were asked if the pharmacist asked them about their knowledge of medication use when filling a prescription. Positive responses were given by over two-thirds of the respondents (67.1%), and 32.9% of the respondents declared a lack of questions of the pharmacist.

The majority of the respondents (57.3%) did not read the medication leaflets, and 42.7% of the respondents always read them.

Most respondents believed labels affixed to medications would definitely facilitate their use (63.4%), 32.9% of the respondents stated that labels were unlikely to facilitate medication use, and 3.7% of the respondents neither agreed, nor disagreed with this statement. None of the respondents had a negative opinion regarding the efficiency of labels, and there was no impact of demographic characteristics or the number of administered medications on their opinions regarding labels efficiency (*p* > 0.05).

The vast majority of the respondents answered positively to the question whether labels with dosing instructions would help in adherence (89%). The remaining 11% neither agreed nor disagreed with this statement. Again, none of the respondents expressed a negative opinion about labels with dosing instructions being helpful in adherence.

Previously received written medical instructions regarding dosing and the method of taking medications could have an influence on the respondents’ opinions. Both the respondents who had and had not received these instructions agreed that labels would be helpful in adherence (*p* > 0.05).

When asked about which medications should have additional labels, the majority of the respondents (67.1%) indicated that it would be reasonable to affix additional labels to all medications, 23.2% indicated prescription medications, and nearly every tenth respondent (9.8%) was of the opinion that labels should be added to over-the-counter medications.

For the majority of the respondents (82.9%), the label system should be included in the pharmaceutical services. One respondent held the opposite opinion (1.2%), and 15.9% of the respondents were indecisive. No dependencies (links) between the willingness to implement labels as a pharmaceutical service and demographic variables or the number of administered medications were found (in both cases *p* > 0.05).

The respondents were also asked if labels should be affixed on patient request. The majority of the respondents gave positive replies (78%), 6.1% were against, and 15.9% had no opinion.

The majority of the respondents (87.8%) were of the opinion that the label system should be available in all pharmacies, 12.2% had no opinion, and none of the respondents were against the implementation of labels in all pharmacies.

## 4. Discussion

The results of this study show that adherence to taking medications regularly and appropriately in chronic diseases (including oncological diseases) is a significant problem. Non-adherence is observed particularly among elderly patients and those taking multiple medicinal products at the same time. The high percentage of elderly people who had problems with adherence can be explained by the many comorbidities in this group, potential problems with memory or dementia.

Non-adherence is observed in a variety of groups of patients, including patients with chronic conditions. Studies show that non-adherence increases with the number of administered medications [16,17,18], which has also been confirmed by the results of our own studies. Another Polish study by Florczak et al. indicates that the percentage of people fully adhering to instructions was just 13.9%, which is slightly higher than in the patient self-evaluation in our own study [19].

In addition to adherence, our own study focused on an evaluation of the respondents’ perception of selected aspects of pharmaceutical care. It was observed that regardless of their health condition or demographic characteristics, the respondents expressed a positive opinion regarding pharmaceutical labels and pharmaceutical consultations.

Pharmacist work is being increasingly transferred to hospital departments, including oncological departments. Considering that a high percentage of hospitalisations result from inappropriately written and used medications [20,21], the role of hospital pharmacists should be expanded by activities connected with admission and establishing pharmacotherapy during and after hospitalisation [22,23,24]. Pharmaceutical counselling would then consist of developing and adjusting standards of medication dispensing and treatment standardisation, which should include the adjustment of pharmacotherapy to the performed medical procedures, as well as individualisation and optimisation of treatment to patient needs and possibilities [25], taking into account their clinical state and the expectations of the comfort of taking medications, their form, route and frequency of administration. The role of pharmacists is also to create a pharmaceutical care plan, which emphasises pharmaceutical consultations [26], good communication and providing information in a clear way. This care may include people with oncological diseases who, due to the multitude of medications used, and additionally possible severe side effects of the drugs used, will require an individual approach, including regular reviews of the drugs used.

When analysing the determinants of pharmaceutical success, it is worth referring to two notions—adherence and compliance [27,28]. The former is defined as the extent to which a patient follows the therapeutic instructions focused on the process or outcomes of treatment. This process means any activities that aim at supervising the course of the disease and include remaining under the control of doctors or specialist outpatient clinics, and taking the recommended medications. If focus is placed on treatment outcomes, curability is perceived as an indicator of therapeutic success. Non-adherence can be intentional or unintentional. Intentional non-adherence occurs when the patient consciously decides to cease or modify the treatment themselves. Unintentional non-adherence can result from a lack of sufficient knowledge on following instructions, or from forgetting to follow them. Compliance refers to the extent to which a patient’s behaviour corresponds to health advice, including pharmacotherapeutic instructions. The key determinants of compliance are the doctor–patient relationships and the cost of treatment [29,30,31].

In the case of chronic patients, the level of adherence depends on a variety of factors that can be divided into five groups: factors associated with the primary disease and comorbidities; factors associated with the treatment; demographic and socioeconomic factors; factors associated with the healthcare system; and factors associated with the patient and their family [4,32,33,34,35]. The same factors may apply to people with cancer.

Thus, the following reasons for medication non-adherence can be listed: long treatment periods, chronic disease; asymptomatic course of a disease; patient does not agree with the diagnosis; polypharmacy; patient does not see treatment outcomes; difficult to remember dosing schemes; and fear of/occurrence of adverse effects.

Attention is also paid to the behavioural aspect of perceiving and following therapeutic instructions. Some experts divide patients in terms of their readiness to adhere to instructions. A lack of cohesion between patient readiness and the treatment strategies suggested by the therapeutic team can indicate a low level of adherence. For this reason, healthcare professionals should be able to regularly evaluate patient readiness to make therapeutic decisions and to adhere to the instructions, as well as to give advice how to properly take part in the therapeutic process. Members of an interdisciplinary team should also monitor patient progress in this respect [36].

In order to help patients take medications properly, instructions should be formulated in a clear and transparent way. One solution is to simplify dosing schemes and, if possible, use sustained release medications, extended half-life medications or therapeutic systems. Labels affixed to medications are of great importance in this respect [37,38], which might be particularly important bearing in mind drug consumption among Polish centenarians [39]. Labels added to prescription medications should contain dosing information and any important warnings related to the product. Inappropriate administration of medications is associated with possible failure of treatment, and in some cases, with health deterioration.

The objective of individual pharmaceutical labels is to increase patient adherence. On the basis of these studies, it was concluded that information on labels should meet agreed standards. It is therefore recommended that the text on pharmaceutical labels be of appropriate size in order to facilitate reading. This information should be concise, and long, extended text should be avoided. Numerals regarding the number of doses or frequency of medication should be presented as figures, and information about the time of medication should be clarified. The most important information should be highlighted in a distinguished font, e.g., small caps. If possible, labels should contain information about the purpose of the medication. Personalised pharmaceutical labels effectively increase patient awareness of their treatment and help reduce problems with remembering to take medications and how to take them [40,41,42].

Upon completion of the study and interpretation of the results, its limitations were evaluated, e.g., a relatively small study group and the fact that patients from only one hospital were taken into consideration. A better picture of the studied issue could be achieved by enrolling patients of other healthcare entities. There were relatively few questions in the questionnaire, which made it impossible to find out about health situation and previous experiences with pharmaceutical care. Although the majority of the respondents perceived various aspects of pharmaceutical care and additional consultations with pharmacists in a positive way, it was not possible to determine their impact on adherence at further stages of their treatment. In the case of further studies, it would be advisable to analyse adherence to pharmaceutical instructions by chronic patients undergoing a two-stage treatment—at both the first visit and during the follow-up.

## 5. Conclusions

Non-adherence is a common phenomenon among patients with oncological diseases. Demographic characteristics that have a significant impact on adherence include age, the kind of work and professional activity. Forgetting to take medications is influenced by the number of long-term medicinal products. This problem is intensified among people taking five or more medications.

Pharmaceutical labels can be an effective method for reducing non-adherence. Oncological patients expressed their willingness to use this type of labels.

## Figures and Tables

**Table 1:** The frequency of forgetting to take medications, taking into account the demographic characteristics of the respondents.

Characteristic	Forgetting to Take Medications				Chi2	*p*
	Often	Sometimes	Seldom	Never		
Sex						
Woman	12.5%	35.4%	37.5%	14.6%	3.66	0.301
Man	20.6%	35.3%	41.2%	2.9%		
Age						
16–19 years	0.0%	0.0%	75.0%	25.0%	42.68	0.000
20–29 years	0.0%	66.7%	0.0%	33.3%		
30–39 years	6.3%	18.8%	50.0%	25.0%		
40–49 years	0.0%	36.8%	57.9%	5.3%		
50–59 years	10.0%	45.0%	40.0%	5.0%		
>60 years	50.0%	40.0%	10.0%	0.0%		
Education						
Primary	27.3%	54.5%	18.2%	0.0%	13.36	0.147
Vocational	25.0%	50.0%	25.0%	0.0%		
Secondary	16.1%	19.4%	51.6%	12.9%		
Higher	7.1%	39.3%	39.3%	14.3%		
Occupation in the sector of/professional activity						
Healthcare	0.0%	0.0%	50.0%	50.0%	43.92	0.000
Intellectual (office) worker	0.0%	40.7%	48.1%	11.1%		
Entrepreneur	10.0%	63.0%	60.0%	0.0%		
Physical worker	19.0%	38.1%	38.1%	4.8%		
Pupil/Student	0.0%	0.0%	50.0%	50.0%		
Retiree/Pensioner	50.0%	43.8%	6.3%	0.0%		
Number of administered medications						
1	0.0%	16.7%	33.3%	50.0%	33.93	0.003
2	12.5%	37.5%	50.0%	0.0%		
3	0.0%	33.3%	50.0%	16.7%		
5	8.3%	54.2%	33.3%	4.2%		
More than 5	41.7%	41.7%	16.7%	0.0%		

## Supplementary Materials

The following supporting information can be downloaded at: https://www.mdpi.com/article/10.3390/ijerph192013642/s1, Figure S1: Pharmacist workflow ROH.
