# Supplementary material for: Implementation of the Patient Counselling Service at the Cancer Hospital in Radom, Poland
Source: Int J Environ Res Public Health. 2022 Oct 21;19(20):13642. doi: 10.3390/ijerph192013642 (PMC9602852; doi:10.3390/ijerph192013642)
Supplement: Supplementary file 1 [file ijerph-19-13642-s001.zip › ijerph-1921374-supplementary.pdf]

HOSPITAL PHARMACY MEDICINES DISPENSING WORKFLOW

REGULAR HOSPITAL PHARMACY PRACTICE

Patient Age 18+

-Information about medicines: patient was only informed about medicines by the doctor, based on what was on the prescription.

-Pharmacist does not provide counselling.

-Pharmacist dispense medicines only and does not provide auxilary labels.

-Pharmacists routine practice is only to fill the prescription from the doctor without any addtional labels or counselling about the patient's treatment nor providing education materials.

↓

-Patient is usually prescribed medicines for 30 days only by the doctor to use at home.
-Nobody checks the patient knowledge at that stage.

↓

-Pharmacist fills the prescription and dispense medicines for 30 days only to use at home.
-Nobody checks the patient knowledge about collected medicines.

HOSPITAL PHARMACY WITH CONSULTATION ROOM AND COUNSELLING

•Patient Age 18+

-Information about medicines: patient counselling included medicine labelling (direction for use, auxilary labels) with pictogram explanation about medicines, instruction how to manage side effects, provision of patient education leaflet included all information in the most simplified form, as well as the instruction how long medicine needs to be used for.
-On the label the pharmacy telephone number was provided in case of any doubts or problems caused by medicines.
-Attached pictogram labels to the medicinal product box included: directions for use, including information when medicine was supposed be taken, potential side effect, warning about interaction with food and other medicines.

↓

- Patient is prescribed medicines for 30 days only to use at home.
- -Pharmacist conduct counselling of the patient whether medicines prescribed where used accordingly to doctor direction.

↓

-Pharmacist fill the prescription and dispense medicines for 30 days only to use at home but also provide counselling: management of side effect, how to reduce side effects, provides details about how to obtain pharmacists support.
-All issues and concernes are being recorded including side effect and drug interactions,
- medicines compliance is being checked and information gathered, including why patients stopped to take medicines
-New medicne batch is being provided including dispensing pictogram labels.

* Explanation of pharmacist counselling service in the oncology hospital in Radom.
